# Positron Emission Tomography Versus Coronary Angiography for Initial Cardiac Screening in Kidney Transplant Candidates: Waitlisting and Clinical Outcomes

**DOI:** 10.7759/cureus.117507

**Published:** 2026-10-05

**Authors:** Mojgan Jalalzadeh, Kevin B Hayes, Jing Shen, Russell J. Pepe, Shareef F. Syed, Kevin Oloomi, Andrew Polyak, Sarfraz Akmal, Steve Khalil, Mital Shah, Zahidul Mondal, Neeraj Singh, Ronald P Pelletier, Advaith Bongu, Leonard Lee

**Affiliations:** 1 Internal Medicine/Nephrology/Transplant, Rutgers Robert Wood Johnson Medical School, New Brunswick, USA; 2 Cardiothoracic Surgery, Rutgers Robert Wood Johnson Medical School, New Brunswick, USA; 3 Medicine and Gastroenterology, Rutgers Robert Wood Johnson Medical School, New Brunswick, USA; 4 Nephrology, Rutgers Robert Wood Johnson Medical School, New Brunswick, USA; 5 Internal Medicine/Nephrology, Rutgers Robert Wood Johnson Medical School, New Brunswick, USA; 6 Internal Medicine/Nephrology/Transplant, Robert Wood Johnson Barnabas Health, New Brunwsick, USA; 7 Internal Medicine, Rutgers Robert Wood Johnson University Hospital, New Brunswick, USA; 8 General Surgery and Transplantation, Robert Wood Johnson Barnabas Health, New Brunswick, USA; 9 Surgery, Robert Wood Johnson Barnabas Health, New Brunswick, USA; 10 Cardiothoracic Surgery, Robert Wood Johnson University Hospital, New Brunswick, USA

**Keywords:** cardiac screening, coronary angiography, kidney transplantation, positron emission tomography, transplant waitlisting

## Abstract

Background

Cardiac evaluation is an important component of kidney transplant evaluation, but the optimal initial testing approach remains uncertain.

Methods

Because the initial test was clinician-selected rather than randomized, this retrospective cohort study examined associations between the initial cardiac testing pathway (PET versus coronary angiography) and transplant listing status among 116 kidney transplant candidates (57 PET, 59 coronary angiography). Because many candidates had already been listed before the index cardiac test, binary analyses were interpreted as associations between clinician-selected testing pathways and listing status rather than post-testing effects on waitlisting. A test-date-anchored landmark analysis, restricted to candidates not yet listed for testing, was used to evaluate subsequent time to listing. Firth-penalized logistic regression and inverse probability of treatment weighting (IPTW) were performed as complementary analyses.

Results

Known coronary artery disease (CAD) differed markedly by testing modality, consistent with confounding by indication and clinician-directed test selection. Overall, 99/116 patients (85.3%) were listed, with no statistically significant difference between groups. Because 31/57 (54.4%) PET patients and 24/59 (40.7%) angiography patients were already listed before testing, the unadjusted evaluation-to-listing timeline could not be attributed to testing modality. The landmark analysis included 58 patients not yet listed at testing, with 55 complete cases in the adjusted model; coronary angiography versus PET was not significantly associated with subsequent time to listing (adjusted hazard ratio [HR], 1.76; 95% CI, 0.76-4.07). In an exploratory subgroup of patients without known CAD (n=30), the estimated rate of subsequent listing was higher with coronary angiography than with PET (HR, 2.58; 95% CI, 1.08-6.16); however, the small subgroup and wide confidence interval warrant caution, and this finding should not be interpreted as evidence of effect modification in the absence of a reproducible formal interaction test. The Firth model showed no significant association between testing pathway and listing status (OR, 1.56; 95% CI, 0.51-4.72), consistent with IPTW (OR, 1.62; 95% CI, 0.54-4.83). Only eight one-year major adverse cardiovascular events occurred, precluding conclusions regarding comparative cardiovascular safety.

Conclusions

Initial cardiac testing was clinician-selected and strongly related to known CAD status; therefore, these findings primarily describe associations between clinically selected testing pathways rather than causal effects of interchangeable testing strategies. After accounting for temporal ordering, no statistically significant overall association between modality and subsequent time to listing was identified. The finding among patients without known CAD was exploratory and requires confirmation. Given the observational design, modest sample size, residual confounding, and limited number of cardiovascular events, these results do not establish the superiority or equivalent safety of either PET or coronary angiography.

## Introduction

Cardiovascular disease is a major cause of morbidity and mortality among patients with end-stage kidney disease and kidney transplant candidates [1-3]. Cardiac risk assessment is therefore an important component of transplant evaluation [3,4]. Current guidance supports noninvasive coronary artery disease (CAD) screening in selected high-risk candidates and emphasizes a risk-stratified approach [3,4]. Importantly, evaluation for previously unknown CAD in asymptomatic candidates differs clinically from assessment of candidates with established CAD, in whom testing may be performed to define coronary anatomy, ischemic burden, or the need for further management.

Available cardiac testing modalities provide different types of information. Positron emission tomography (PET) myocardial perfusion imaging provides a noninvasive functional assessment. It can quantify myocardial blood flow and coronary flow reserve, although its role as a first-line approach in kidney transplant candidates remains incompletely defined [2-6]. Coronary angiography provides direct anatomic assessment and permits revascularization when clinically indicated [3,7,8]. However, routine invasive evaluation of asymptomatic patients with advanced kidney disease remains controversial [3,4,9].

In clinical practice, PET and coronary angiography are not necessarily interchangeable initial strategies. In our center, PET was generally used as the initial noninvasive test when there was no clear indication for immediate angiography. In contrast, coronary angiography was preferentially used when clinically indicated, including in candidates with known CAD, high pretest probability, or a cardiology recommendation. Accordingly, comparisons between these modalities may reflect differences in clinical indication and baseline cardiovascular risk, as well as any association with subsequent transplant evaluation outcomes.

Evidence directly examining the relationship between PET and coronary angiography, as well as kidney transplant waitlisting, remains limited [3-6,10]. Therefore, the primary objective of this retrospective cohort study was to evaluate the association between clinician-selected initial cardiac testing pathway (PET versus coronary angiography) and subsequent kidney transplant waitlisting among adult transplant candidates, using a test-date landmark analysis as the primary time-to-event approach to account for reverse temporal ordering. Secondary objectives were to explore associations within strata defined by known CAD status and to describe cardiovascular outcomes following the initial cardiac evaluation. Because the testing modality was clinician-selected based on clinical indication rather than randomly assigned, these analyses were intended to characterize associations between clinical testing pathways and outcomes rather than to compare the effectiveness of interchangeable testing strategies.

## Materials and methods

Ethics approval

This study was approved by the Institutional Review Board of Rutgers Robert Wood Johnson Medical School (IRB # Pro2025002041); informed consent was waived due to the retrospective design. The study adhered to the Declaration of Helsinki and was reported in accordance with the Strengthening the Reporting of Observational Studies in Epidemiology (STROBE) guidelines.

Study design, population, and exposure

This retrospective cohort study included adult patients (aged 18 years or older) who underwent kidney transplant evaluation, including cardiac evaluation, at Robert Wood Johnson University Hospital between January 2022 and December 2024. Patients were excluded if a transplant was completed before their cardiac evaluation, if the initial cardiac testing modality was undocumented, or if waitlisting outcome data were missing; no patients met the pre-evaluation transplant exclusion, and post-listing transplantation was analyzed as a study outcome rather than an exclusion criterion. For patients with more than one eligible evaluation, the earliest qualifying evaluation served as the index date. The patient selection flow is shown in Figure 1.

**Figure 1:**
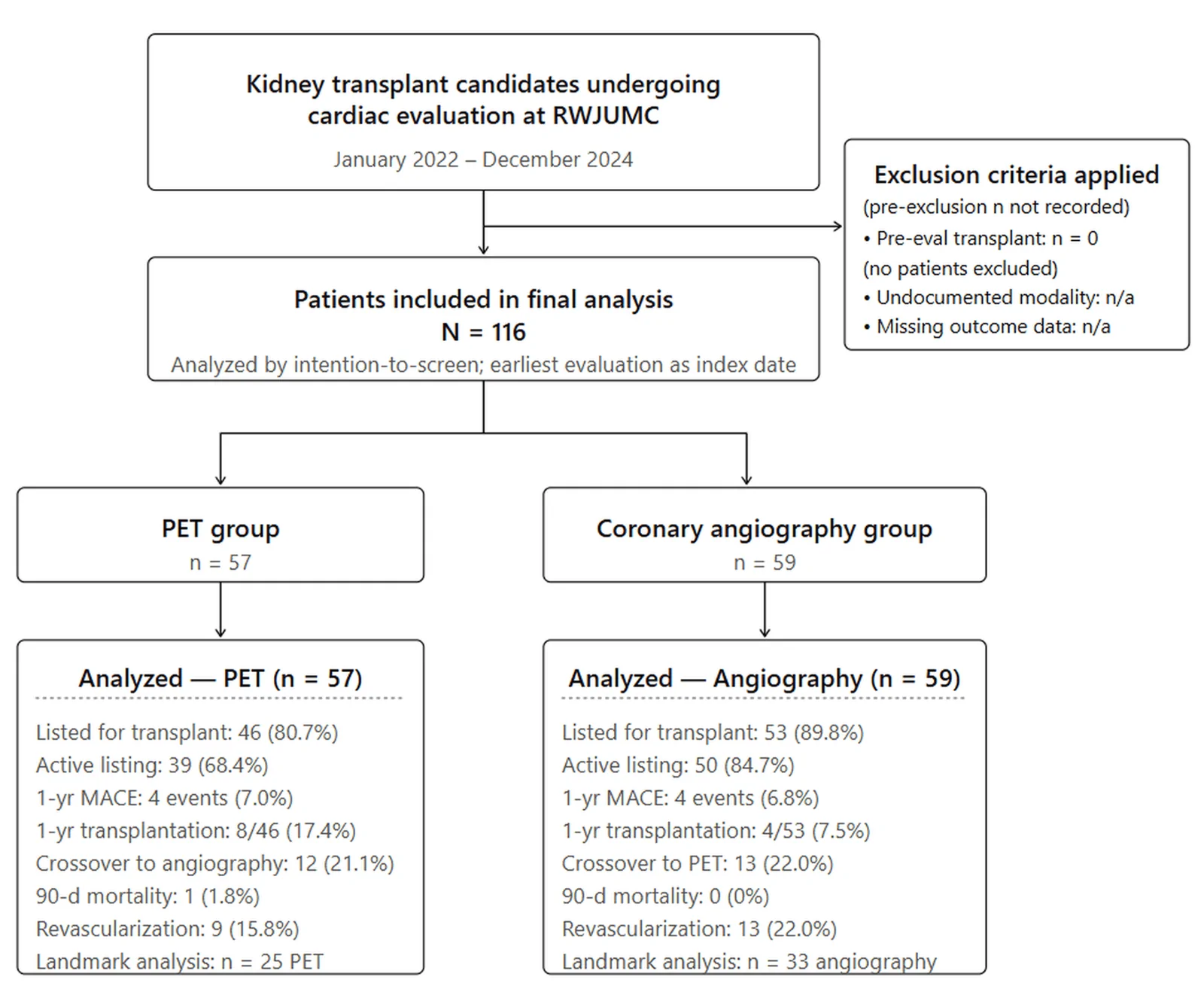
STROBE Flow Diagram of Study Participants. The diagram summarizes participant selection, exclusions, initial cardiac screening modality, and key study outcomes among kidney transplant candidates. STROBE = Strengthening the Reporting of Observational Studies in Epidemiology; PET = positron emission tomography; MACE = major adverse cardiovascular events; RWJUMC = Robert Wood Johnson University Hospital; n/a = number not separately recorded in source data. Landmark analysis restricted to patients not yet listed at cardiac test date.

Exposure was defined as the initial cardiac test (PET or coronary angiography) and was classified by the first test performed, regardless of subsequent crossover to the alternative modality. During the study period, PET was generally used as the initial noninvasive test absent a clear indication for immediate angiography; an abnormal or high-risk PET result prompted cardiology review and consideration of angiography, which was used as the initial modality when clinically indicated (e.g., known CAD, high pretest probability, or cardiology recommendation).

Variables and outcomes

Clinical and demographic data were extracted from the electronic medical record and transplant databases, including age, sex, race, body mass index, dialysis duration, diabetes, hypertension, hyperlipidemia, smoking history, CAD, prior coronary stenting, left ventricular ejection fraction (LVEF), estimated glomerular filtration rate (eGFR), peripheral artery disease, and cardiovascular events. LVEF was obtained from the imaging study closest to the evaluation; coronary anatomy variables were collected descriptively but excluded from regression models due to inconsistent availability before the initial test-selection decision. The primary outcome was transplant waitlisting; secondary outcomes included time to listing, active listing, United Network for Organ Sharing (UNOS) qualification, one-year transplantation, and cardiovascular outcomes (90-day mortality, one-year major adverse cardiovascular events (MACE) and its components, heart failure, revascularization).

Waitlisting outcomes

The primary outcome was kidney transplant waitlisting as recorded in the transplant database, including candidates who had been placed on the waitlist but remained inactive (Status 7-type). Active listing was therefore analyzed separately as a secondary and sensitivity outcome, since inactive listing may precede completion of cardiac clearance. A gap existed between listing and active-listing dates for 20/46 (43.5%) of PET and 20/53 (37.7%) of angiography patients. "UNOS qualification" was a distinct field, generally reflecting the Organ Procurement and Transplantation Network (OPTN)waiting-time eligibility rather than the date of listing itself; however, because UNOS qualification dates were recorded exclusively for candidates formally listed at this center, the UNOS-qualified and listed counts were identical (46 PET, 53 angiography).

Because this was a retrospective analysis of previously abstracted transplant data, the specific administrative event represented by the recorded waitlist date, and the abstraction rule applied when multiple listing-related dates existed, could not be determined with certainty from the available data. We therefore did not impose an unverified date hierarchy retrospectively. Three angiography patients were coded as listed but lacked a documentable listing date on chart review; these patients were retained as listed in binary outcome analyses but excluded from analyses requiring an exact listing date. At least one nonlisted patient was later found to have been listed and transplanted at another center, indicating that outside-center listings were not systematically captured in this single-center dataset.

Statistical analysis

Continuous variables were summarized as mean ± SD or median (IQR) and compared using the t-test or Wilcoxon rank-sum test, as appropriate; categorical variables were compared using the chi-square test or Fisher's exact test when expected cell counts were smaller than 10; standardized mean differences (SMD) were used to assess covariate balance, particularly before and after propensity-score weighting. Because a substantial proportion of candidates had already been listed before their index cardiac test, analyses using the transplant evaluation date as time zero could not validly estimate subsequent waitlisting associated with testing modality. Accordingly, the primary time-to-event analysis was a test-date landmark analysis, restricted to candidates who had not yet been listed at the time of their index cardiac test, with the index cardiac test date defined as time zero. Kaplan-Meier estimates, log-rank testing, and Cox proportional hazards regression were used to evaluate subsequent time to listing. Analyses based on evaluation-to-listing intervals were considered descriptive and were not interpreted as post-testing effects. We also stratified the primary comparison by known versus unknown CAD status, identified a priori as a principal driver of test selection (Figure 2).

**Figure 2:**
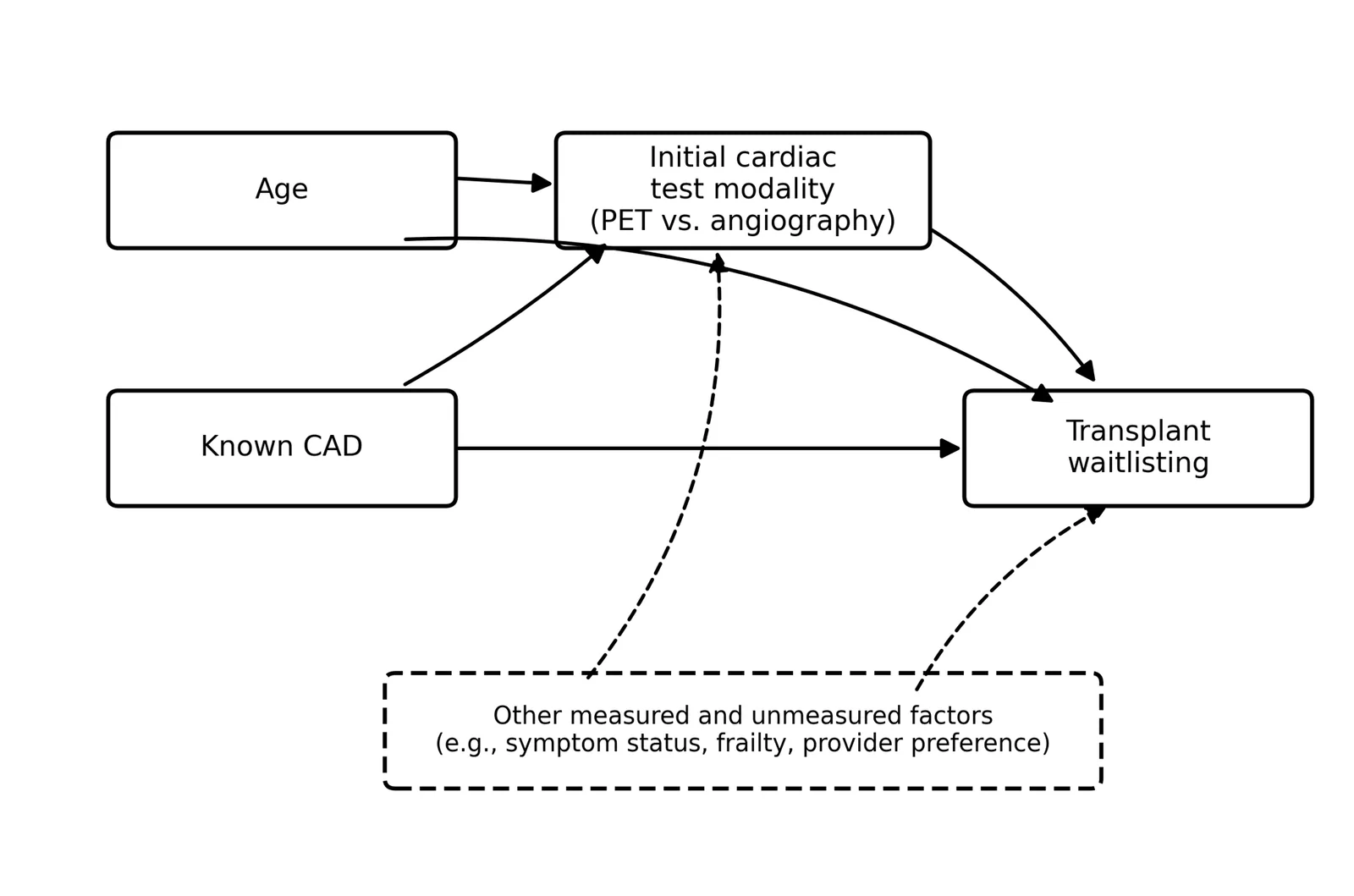
Directed Acyclic Graph Illustrating the Assumed Causal Structure Used to Identify the Reduced Adjustment Set. Age and known coronary artery disease (CAD) were considered the principal measured shared causes of clinician-selected initial cardiac testing modality and transplant waitlisting. The diagram represents the causal assumptions underlying the reduced adjustment set used in the primary Firth-penalized logistic model, the test-date landmark Cox model, and the inverse probability of treatment weighting sensitivity analysis. Other measured and unmeasured factors, such as symptom status, frailty, and provider preference, may contribute to test selection and waitlisting and are a source of residual confounding not captured by the reduced adjustment set. PET = positron emission tomography.

For adjusted analyses, a larger eight-covariate model, including age, sex, dialysis duration, diabetes mellitus, known CAD, LVEF, smoking history, and peripheral artery disease, was initially considered but not retained because the small number of non-listing events produced quasi-complete separation and unstable parameter estimates. A reduced adjustment set consisting of age and known CAD was therefore used for the primary Firth-penalized logistic model. The primary test-date landmark Cox model was adjusted for age, known CAD, and LVEF and included 55 candidates with complete data for these covariates. Age and known CAD were selected for the reduced adjustment set based on the assumed causal structure represented in a directed acyclic graph (DAG) (Figure 2).

In that DAG, age and known CAD were considered the principal measured shared causes of testing modality and waitlisting, rather than being selected based on statistical significance or post-weighting covariate balance.

Because an odds ratio can differ substantially from risk ratios and risk differences when the outcome is common, we additionally computed model-standardized adjusted probabilities, absolute risk difference, and risk ratio, and performed a nonparametric bootstrap analysis using 2,000 resamples to obtain confidence intervals for the model-derived effect estimates.

Inverse probability of treatment weighting (IPTW) targeting the average treatment effect was performed as a sensitivity analysis. A propensity-score model incorporating all 12 candidate covariates failed to converge and showed limited overlap in the propensity score across some covariate strata (e.g., race). We therefore used the same reduced propensity model (age and known CAD) for the IPTW sensitivity analysis. We acknowledge that this reduced model did not balance all measured covariates: residual imbalance remained after weighting for LVEF and dialysis duration, among other variables. We assessed positivity and propensity-score overlap using propensity-score distributions and ranges, summarized the weight distribution and extreme weights, calculated effective sample size (ESS), and assessed covariate balance before and after weighting using robust standard errors for weighted models. We repeated weighted analyses after truncating weights at the 1st/99th and 5th/95th percentiles. To examine sensitivity to residual imbalances in LVEF and dialysis duration, we additionally fitted a doubly robust model that adjusted for these two covariates, yielding a similar waitlisting estimate. Because the reduced models cannot eliminate residual measured or unmeasured confounding, their estimates were interpreted as adjusted associations rather than causal effects. Three additional sensitivity analyses excluded crossover patients, reclassified patients using an “ever coronary angiography” approach, and replaced negative time-to-event values with zero. Variables with <5% missingness were analyzed using complete cases. Dialysis duration, which had 21/116 (18.1%) missing observations, was analyzed using multiple imputation by chained equations, with results compared with complete-case analyses. The number of imputed datasets, the variables included in the imputation model, the iteration count, and the pooling method used to combine estimates were not recorded in the retained analysis materials and could not be verified for reporting. Given the retrospective observational design and the fixed sample size, no formal a priori sample size calculation was performed. Analyses were performed using R version 4.3.2 (R Foundation for Statistical Computing, Vienna, Austria), with two-sided tests and p<0.05 considered statistically significant. For inferential comparisons that could be reliably reproduced from the archived analytic dataset, the corresponding test statistic (t, χ², or the Mann-Whitney U statistic) and degrees of freedom (t, χ²) are provided in the associated table, together with an appropriate effect size (phi coefficient or Cramér's V for chi-square comparisons); tables reporting regression-based estimates provide the odds ratio or hazard ratio with its 95% confidence interval in place of a separate test statistic, consistent with standard reporting for regression-based estimates.

## Results

Study cohort and analytic samples

The full cohort included 116 kidney transplant candidates, of whom 57 underwent PET and 59 underwent coronary angiography as the initial cardiac test. Analytic sample sizes varied based on the availability of outcomes and model requirements. The reduced Firth regression included 110 candidates with complete data for the variables included in that model (age and known CAD). Among the listed candidates, 96 had a documented listing date available for analyses requiring an exact event date. Candidates recorded as listed but lacking a documentable listing date were retained in binary listing analyses but excluded from analyses requiring an exact listing date. The primary landmark analysis was restricted to the 58 candidates who had not yet been listed at the time of their index cardiac test (25 PET and 33 angiography); 55 had complete covariate data for the adjusted Cox model.

Study population and baseline characteristics

Between January 2022 and December 2024, 116 patients underwent cardiac evaluation for kidney transplant (57 PET, 59 angiography), and the results were analyzed according to the initial cardiac test performed (Table 1). Mean age was 59.9±8.8 years (PET) versus 56.7±9.5 years (angiography; p=0.072). Male sex, race, dialysis duration, diabetes, and smoking history were broadly similar between groups. LVEF was higher with PET (60.5±6.3% vs. 55.0±11.0%; SMD=0.61; p=0.002), and CAD was more common with angiography (20/57, 35.1% vs. 37/59, 62.7%; SMD=0.59; p=0.005), with a correspondingly higher affected-vessel count (median 0 vs. 1; SMD=0.95; p<0.001); prior coronary stenting was similar (12/57, 21.1% vs. 13/59, 22.0%).

**Table 1:** Baseline Characteristics by Initial Cardiac Evaluation Modality Continuous variables are presented as mean ± SD or median (IQR), depending on distribution. Categorical variables are presented as n (%). Approximately normally distributed continuous variables (age, body mass index, LVEF, eGFR) were compared using the independent-samples t-test (reported as t(df)); non-normally distributed continuous variables (dialysis duration, pack-years, number of affected vessels) were compared using the Wilcoxon rank-sum test (Mann-Whitney U statistic); categorical variables were compared using the chi-square test or Fisher’s exact test (reported as χ²(df), with phi coefficient (φ) or, for race, Cramér's V, as a measure of effect size). *For dialysis duration, the test statistic and corresponding P value could not be reliably reproduced from the archived analytic dataset and, therefore, are not reported. SMD = standardized mean difference; absolute SMD values <0.1 between groups were considered indicative of acceptable covariate balance; PET = positron emission tomography; LVEF = left ventricular ejection fraction; eGFR = estimated glomerular filtration rate; IQR = interquartile range.

Variable	PET (n = 57)	Coronary angiography (n = 59)	SMD	p-value	Test statistic	Effect size
Age, years, mean ± SD	59.9 ± 8.8	56.7 ± 9.5	0.35	0.072	t(110) = 1.82	N/A
Male sex, n (%)	36 (63.2)	40 (67.8)	0.10	0.741	χ²(1) = 0.11	φ = 0.03
Race, n (%)	N/A	N/A	0.35	0.508	χ²(4) = 3.30	V = 0.17
White	23 (40.4)	15 (25.4)	N/A	N/A	N/A	N/A
Black	17 (29.8)	24 (40.7)	N/A	N/A	N/A	N/A
Hispanic	8 (14.0)	9 (15.3)	N/A	N/A	N/A	N/A
Asian	7 (12.3)	8 (13.6)	N/A	N/A	N/A	N/A
Other	1 (1.8)	2 (3.4)	N/A	N/A	N/A	N/A
Body mass index (kg/m²), mean ± SD	30.9 ± 6.3	30.0 ± 5.8	0.16	0.393	t(111) = 0.86	N/A
Dialysis duration, months, median (IQR)	33.3 (13.1, 40.2)	17.2 (6.8, 33.8)	0.13	—*	NA	NA
Comorbidities						
Diabetes mellitus, n (%)	47 (82.5)	45 (76.3)	0.13	0.661	χ²(1) = 0.19	φ = 0.04
Hypertension, n (%)	55 (96.5)	55 (93.2)	0.11	1.000	χ²(1) = 0.00	φ = 0.00
Hyperlipidemia, n (%)	31 (54.4)	34 (57.6)	0.09	0.786	χ²(1) = 0.07	φ = 0.03
Smoking history, n (%)	17 (29.8)	22 (37.3)	0.19	0.428	χ²(1) = 0.63	φ = 0.07
Pack-years (among ever-smokers), median (IQR)	15 (12, 40)	15 (9.7, 26.4)	0.33	0.509	U = 89.5	N/A
Cerebrovascular disease, n (%)	12 (21.2)	8 (13.6)	0.19	0.459	χ²(1) = 0.55	φ = 0.07
Peripheral artery disease, n (%)	2 (3.5)	3 (5.1)	0.08	1.000	χ²(1) = 0.00	φ = 0.00
Coronary artery disease, n (%)	20 (35.1)	37 (62.7)	0.59	0.005	χ²(1) = 7.90	φ = 0.26
LVEF, %, mean ± SD	60.5 ± 6.27	55.0 ± 11.0	0.61	0.002	t(111) = 3.23	N/A
eGFR, mL/min/1.73 m², mean ± SD	10.3 ± 4.77	9.8 ± 4.0	0.12	0.527	t(111) = 0.66	N/A
Coronary anatomy (descriptive only)						
Number of affected vessels, median (IQR)	0 (0, 0)	1 (0, 3)	0.95	<0.001	U = 701.0	N/A
Prior coronary stenting, n (%)	12 (21.1)	13 (22.0)	0.02	1.000	χ²(1) = 0.00	φ = 0.00

Primary outcome: waitlisting

Overall, 99 of 116 patients (85.3%) were listed: 46 of 57 patients (80.7%) in the PET group and 53 of 59 patients (89.8%) in the angiography group (p=0.237). The binary listed (yes/no) variable was the primary outcome for all binary analyses, including the descriptive comparison in Table 2, the Firth regression model (Table 3), and the IPTW analysis. Three angiography patients were coded as listed (Y=1) in the source data but lacked a documentable listing date on chart review; these three patients were retained as listed in all binary analyses and the regression model, consistent with the primary outcome definition. They were excluded only from analyses requiring an exact listing date, specifically, time-to-event analyses, for which 96 patients had a documented listing date. Accordingly, the Firth model (Table 3) and IPTW analysis reflect a listed count of 94 in the complete-case sample (99 listed minus 5 with missing covariate data), and all time-to-event analyses use the 96 patients with confirmed listing dates. A full 8-covariate logistic model (age, sex, dialysis duration, diabetes mellitus, CAD, LVEF, smoking history, and peripheral artery disease) showed quasi-complete separation (n=90 complete cases, 16 non-listed) and was not used for inference. In the reduced Firth model (age, known CAD; n=110), angiography showed a nonsignificant, imprecise association with listing (aOR, 1.56; 95% CI, 0.51-4.72; p=0.43) (Table 3). The model-standardized probability of listing was 87.0% with angiography versus 81.2% with PET (absolute risk difference, 5.8 percentage points; risk ratio, 1.07), indicating the odds ratio overstates the absolute effect; bootstrap results were concordant (OR 95% CI, 0.58-4.70; risk difference 95% CI, -6.2 to 18.5 points).

**Table 2:** Transplant Evaluation and Waitlisting Outcomes by Initial Cardiac Evaluation Modality Time-to-event variables are reported as median (IQR). Categorical variables are reported as n (%). Comparisons were performed using the Wilcoxon rank-sum test (Mann-Whitney U statistic) for continuous variables and the chi-square test or Fisher’s exact test (reported as χ²(df), with phi coefficient (φ) as effect size) for categorical variables. *Evaluation-date interval comparisons (evaluation to cardiac study, evaluation to listing, evaluation to active listing, and active listing) are presented descriptively because the index cardiac test occurred after listing in a substantial proportion of candidates. Inferential P values that could not be reliably reproduced from the archived analytic dataset were omitted (—). A test-date landmark approach was used for the primary time-to-event analysis. PET = positron emission tomography; IQR = interquartile range; SMD = standardized mean difference; UNOS = United Network for Organ Sharing.

Outcome	PET (n = 57)	Coronary angiography (n= 59)	SMD	p-value	Test statistic	Effect size
Listed for transplant, n (%)	46 (80.7)	53 (89.8)	0.26	0.237	χ²(1) = 1.40	φ = 0.11
Time from evaluation to cardiac study, days, median (IQR)	140.5 (37.8, 508.5)	213.5 (110.5, 437)	0.16	—*	NA*	NA*
Time from evaluation to listing, days, median (IQR)	64 (17, 261)	193.5 (33.5, 322.8)	0.50	—*	NA*	NA*
Time from evaluation to active listing, days, median (IQR)	281 (217.3, 574.3)	318.5 (234.8, 626)	-0.01	—*	NA*	NA*
Actively listed, n (%)	39 (68.4)	50 (84.7)	0.39	—*	NA*	NA*
UNOS qualified, n (%)	46 (80.7)	53 (89.8)	0.26	0.237	χ²(1) = 1.40	φ = 0.11

**Table 3:** Firth-Penalized Logistic Regression for Transplant Waitlisting A full 8-covariate logistic model (age, sex, dialysis duration, diabetes mellitus, known CAD, LVEF, smoking history, peripheral artery disease) showed quasi-complete separation (n=90 complete cases, 16 non-listed); given this instability, it was not used for inference and is not interpreted as a primary result. The outcome was listed for kidney transplantation. Odds ratios were estimated using Firth-penalized logistic regression with a reduced covariate set (age and known CAD), selected based on a directed acyclic graph that identified these as the principal shared causes of both testing modality and waitlisting. PET was the reference modality. This table presents the complete-case analytic sample for this model: 110 of 116 candidates with complete data for age and known CAD. The model-standardized probability of listing was 87.0% with angiography versus 81.2% with PET (absolute risk difference, 5.8 percentage points; risk ratio, 1.07); bootstrap 95% CIs (2000 resamples) were 0.58-4.70 for the odds ratio and -6.2 to 18.5 percentage points for the risk difference. CI = confidence interval; CAD = coronary artery disease; LVEF = left ventricular ejection fraction; PET = positron emission tomography.

Variable	Not listed (N=16)	Transplant listing (N=94)	Odds ratio	95% CI	p-value
Initial cardiac testing modality (coronary angiography vs. PET), n/N (%)	7/16 (43.8)	48/94 (51.1)	1.56	0.51-4.72	0.434
Age (per year), mean ± SD	59.2 ± 7.9	58.0 ± 9.6	1.00	0.94-1.05	0.858
Coronary Artery Disease (CAD), n/N (%)	10/16 (62.5)	46/94 (48.9)	0.53	0.17-1.60	0.259

A structured reason for nonlisting was not captured; free-text notes (16 of 17 patients) most often cited incomplete cardiac workup (10 patients) rather than a specific contraindication; one cited uncontrolled hypertension; and three reflected an unexplained committee decision. One non-listed patient was later found listed and transplanted at another center.

Evaluation and listing timelines

Median time from evaluation to cardiac study was 140.5 days (IQR, 37.8-508.5) in the PET group and 213.5 days (IQR, 110.5-437) in the angiography group. Median time from evaluation to listing was 64 days (IQR, 17-261) in the PET group and 193.5 days (IQR, 33.5-322.8) in the angiography group, and median time from evaluation to active listing was 281 days (IQR, 217.3-574.3) versus 318.5 days (IQR, 234.8-626), respectively; active listing was achieved in 39/57 (68.4%) versus 50/59 (84.7%) of PET and angiography patients. These evaluation-date interval comparisons were considered descriptive because many candidates had already been listed before their index cardiac test, and the corresponding p-values could not be reliably reproduced from the archived analytic dataset (Table 2). UNOS qualification was achieved in 46/57 (80.7%) versus 53/59 (89.8%) (p=0.237).

Temporal relationship between testing and listing

Because the evaluation date served as the time origin above, but the subsequent index test defined the exposure, we examined temporal ordering: 31/57 (54.4%) of PET and 24/59 (40.7%) of angiography patients were listed before their index test, precluding valid attribution of the unadjusted timeline to modality. In a landmark analysis restricted to the 58 patients (25 PET, 33 angiography) unlisted as of testing, median time from test to listing was 181 days (PET) versus 162 days (angiography), with no significant difference (log-rank p=0.96; unadjusted HR, 0.98; adjusted HR, 1.76 (95% CI, 0.76-4.07; p=0.19; n=55)) (Table 4, Figure 3). CAD was independently associated with a lower hazard of listing (aHR, 0.29; 95% CI, 0.13-0.64; p=0.002), consistent with the notion that additional workup delays listing. These findings indicate that the apparent unadjusted PET advantage was substantially affected by reverse temporal ordering and should not be interpreted as evidence of a modality effect.

**Table 4:** Test-Date-Anchored Cox Proportional Hazards Model for Time to Waitlisting (Landmark Analysis) This test-date-anchored landmark analysis (Results: Temporal Relationship Between Cardiac Testing and Listing) was restricted to the 58 patients who had not been listed before or at the time of their index cardiac test (25 PET, 33 angiography; 55 with complete covariate data for this adjusted model). Time zero is the index cardiac test date, not the evaluation date. PET is the reference modality for initial cardiac testing. CAD = coronary artery disease; CI = confidence interval; LVEF = left ventricular ejection fraction; PET = positron emission tomography.

Variable	Listing events, PET (n)	Listing events, Coronary angiography (n)	Person-Days at Risk, PET	Person-Days at Risk, Coronary angiography	Hazard ratio	95% CI	p-value
Initial cardiac testing modality (coronary angiography vs. PET)	25	15	5587	3120	1.76	0.76-4.07	0.19
Age (per year)	-	-	-	-	1.00	0.96-1.04	0.99
Coronary Artery Disease (CAD, Yes vs. No)	-	-	-	-	0.29	0.13-0.64	0.002
LVEF (per %)	-	-	-	-	1.00	0.96-1.05	0.98

**Figure 3:**
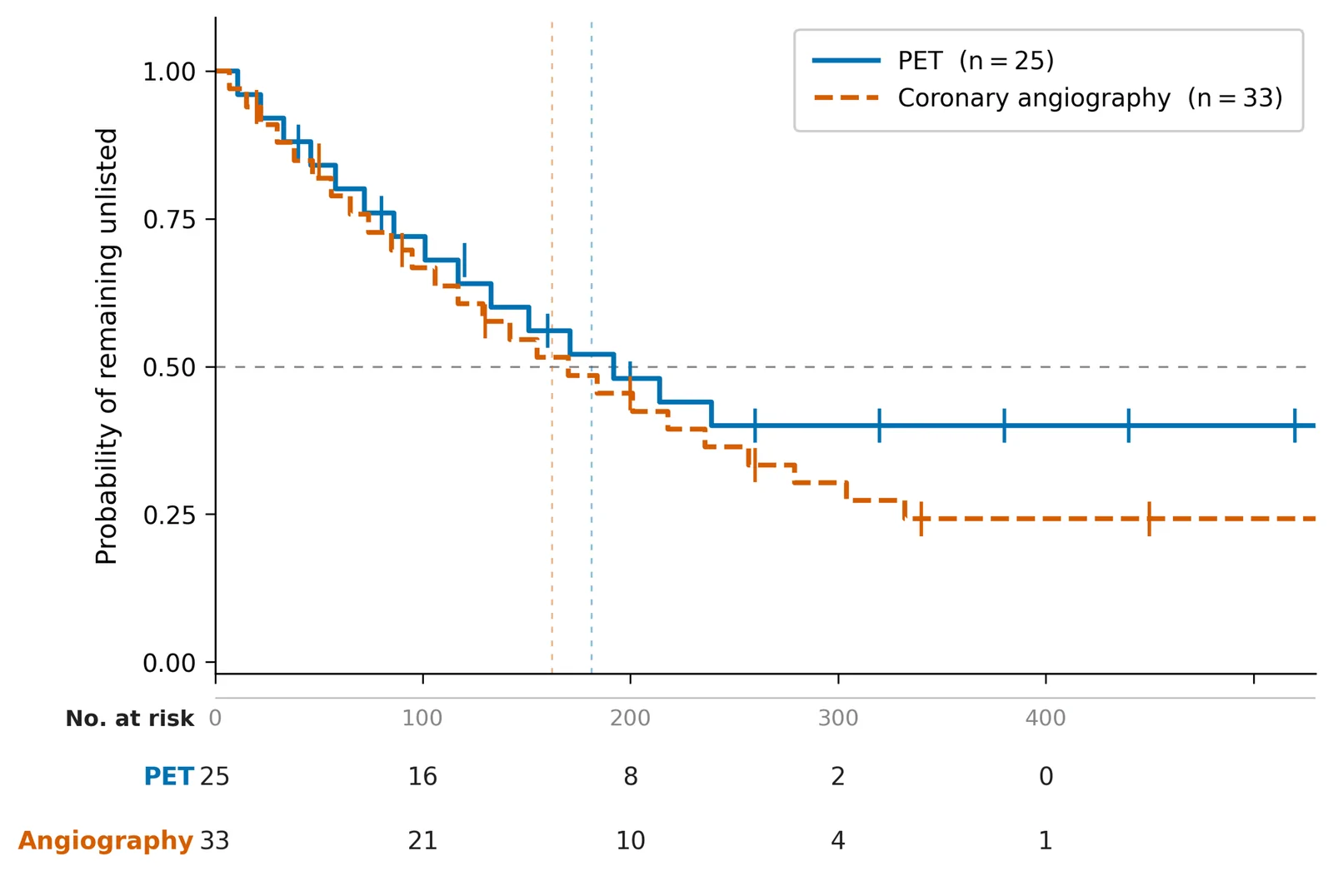
Kaplan-Meier Curves for Time From Index Cardiac Test to Listing (Landmark Analysis, At-Risk Cohort). Patients were restricted to the 58 candidates who were not yet listed at the time of their index cardiac test (25 PET, 33 coronary angiography), with the test date used as time zero. The solid blue line represents the PET group (n = 25), and the dashed orange line represents the coronary angiography group (n = 33). Tick marks indicate censored observations. The dashed horizontal line indicates S(t) = 0.50, and the dashed vertical lines indicate the observed median times to waitlisting (162 days for coronary angiography and 181 days for PET). The unadjusted log-rank p-value was 0.96. The adjusted hazard ratio for coronary angiography versus PET was 1.76 (95% CI, 0.76-4.07; p = 0.19) in a Cox proportional hazards model adjusted for age, coronary artery disease status, and left ventricular ejection fraction (n = 55 complete cases). PET = positron emission tomography.

Active listing sensitivity analysis

Because inactive listing can precede completed cardiac clearance, we repeated key analyses using active listing (achieved in 39/57 (68.4%) of PET and 50/59 (84.7%) of angiography). Reverse temporal ordering was less frequent with this endpoint (11/56, 19.6% PET; 6/57, 10.5% angiography active-listed before testing, versus 31/57, 54.4% and 24/59, 40.7% for any listing). Angiography showed higher, borderline odds of active listing (OR, 2.24; 95% CI, 0.90-5.58; p=0.085). A landmark analysis (n=96: 45 PET, 51 angiography) showed no significant association between modality and time to active listing (HR, 0.64; 95% CI, 0.37-1.11; p=0.11, expressed as PET versus angiography); that is, PET patients reached active listing more slowly than angiography patients, directionally consistent with the any-listing landmark result, in which angiography was associated with faster listing (Table 4).

Exploratory analysis by known CAD status

Coronary artery disease-stratified landmark analyses were exploratory. Known CAD differed markedly by modality: among no-known-CAD patients (n=56), 35/56 (62.5%) underwent PET; among known-CAD patients (n=57), 37/57 (64.9%) underwent angiography, confirming indication-based rather than random test selection. Three patients could not be classified for the stratified analysis and were excluded. This subgroup classification could not be fully reconciled with the baseline descriptive coding in the archived dataset, and no further assumptions regarding the CAD status of these three patients were made. Among candidates without known CAD, the landmark cohort included 30 patients (18 PET and 12 angiography), and the estimated rate of subsequent listing was higher with coronary angiography than with PET (HR, 2.58; 95% CI, 1.08-6.16). Given the small subgroup size and wide confidence interval, this finding should be interpreted with caution. Among candidates with known CAD, the landmark cohort included 26 patients (6 PET and 20 angiography), and no statistically significant association was observed (HR, 0.96; 95% CI, 0.26-3.50), though the small PET subgroup limited this estimate. Prior coronary stenting did not differ meaningfully between groups and did not appear to explain these subgroup differences. Statistical significance within one subgroup but not the other does not establish effect modification. A formal modality-by-CAD interaction analysis could not be reliably reconstructed from the archived analytic dataset; therefore, these exploratory subgroup findings should not be interpreted as evidence that known CAD status modifies the association between testing modality and subsequent listing.

Cardiovascular and transplant outcomes

Ninety-day mortality (1/57, 1.8% vs. 0/59, 0%) and one-year MACE (4/57, 7.0% vs. 4/59, 6.8%; p=1.000) showed no statistically detectable difference between groups, including individual components (myocardial infarction, heart failure, cerebrovascular accident, cardiovascular death) and revascularization (9/57, 15.8% vs. 13/59, 22.0%; p=0.677) (Table 5); however, only eight one-year MACE events occurred overall, so the study was underpowered for cardiovascular outcomes and equivalent safety cannot be concluded. Among listed patients, one-year transplantation occurred in 8/46 (17.4%) (PET) versus 4/53 (7.5%) (angiography; p=0.264), with similar times from listing to transplant (292 vs. 440 days) and from intervention to transplant (123 vs. 233 days) (Table 6).

**Table 5:** Cardiovascular Outcomes by Initial Cardiac Evaluation Modality ᵃ MACE was defined as a composite of myocardial infarction, cerebrovascular accident, or cardiovascular death. Heart failure hospitalization and coronary revascularization were not included in the MACE composite and are reported as separate cardiovascular outcomes. Revascularization performed at the time of the index angiogram was excluded from the revascularization outcome; only revascularization occurring after the index evaluation was included. Atrial fibrillation was not included in the MACE composite and is reported separately as an exploratory outcome. Categorical variables were compared using the chi-square test or Fisher’s exact test (reported as χ²(df), with phi coefficient (φ) as effect size). PET = positron emission tomography; MACE = major adverse cardiovascular events; SMD = standardized mean difference.

Outcome	PET (n = 57)	Coronary angiography (n = 59)	SMD	p-value	Test statistic	Effect size
90-day all-cause mortality, n (%)	1 (1.8)	0 (0)	0.19	0.993	χ²(1) = 0.00	φ = 0.00
1-year MACEᵃ, n (%)	4 (7.0)	4 (6.8)	0.00	1.000	χ²(1) = 0.00	φ = 0.00
Myocardial infarction	3 (5.3)	3 (5.1)	0.00	1.000	χ²(1) = 0.00	φ = 0.00
Heart failure hospitalization	3 (5.3)	7 (11.9)	0.25	0.335	χ²(1) = 0.93	φ = 0.09
Cerebrovascular accident	0 (0)	1 (1.7)	0.19	1.000	χ²(1) = 0.00	φ = 0.00
Cardiovascular death	1 (1.8)	0 (0)	0.19	0.993	χ²(1) = 0.00	φ = 0.00
Coronary revascularizationᵃ	9 (15.8)	13 (22.0)	0.13	0.677	χ²(1) = 0.17	φ = 0.04
Exploratory outcome						
New-onset atrial fibrillation, n (%)	1 (1.8)	3 (5.1)	0.19	0.623	χ²(1) = 0.24	φ = 0.05

**Table 6:** Post-listing Transplant Outcomes by Initial Cardiac Evaluation Modality Time-to-event variables are reported as median (IQR). Categorical variables are reported as n (%). The denominator for 1-year transplantation after listing is restricted to patients who were formally listed (PET: n = 46; coronary angiography: n = 53). The categorical outcome was compared using the chi-square test or Fisher’s exact test (reported as χ²(df), with phi coefficient (φ) as effect size); time-to-event outcomes were compared using the Wilcoxon rank-sum test. *For the two time-to-transplant comparisons, the small subgroup sizes precluded reliable recomputation of the test statistic from the archived analytic dataset; the corresponding inferential P values were therefore omitted (—) rather than retained without a verifiable recomputed statistic. ᵃDenominator restricted to patients who were listed for transplantation (n=46 PET, n=53 angiography), since this outcome is only clinically defined among listed patients. ᵇAmong patients who underwent cardiac intervention and were subsequently transplanted. PET = positron emission tomography; IQR = interquartile range; SMD = standardized mean difference.

Outcome	PET (n = 46 Listed)	Coronary angiography (n = 53 Listed)	SMD	P-value	Test statistic	Effect size
Transplanted within 1 year of listing, n (%)^a^	8 (17.4)	4 (7.5)	0.29	0.264	χ²(1) = 1.25	φ = 0.11
Time from listing to transplant, days, median (IQR)	292 (133, 424)	440 (87, 931)	0.07	—*	NA*	NA*
Time from cardiac intervention to transplant, days, median (IQR)^b^	123 (95, 284)	233 (123, 500)	0.74	—*	NA*	NA*

Propensity score-weighted analysis

The original 12-covariate propensity model did not converge (due to limited overlap, notably by race). Using the reduced model (age, known CAD; n=110), propensity scores indicated adequate overlap. Age and CAD balanced almost exactly after weighting; LVEF (SMD, 0.37), hypertension (0.22), and dialysis duration (0.33) remained imbalanced (Table 7, Figure 4). Stabilized weights had a median of 0.85 (IQR, 0.73-1.17; 1st/99th percentiles, 0.59/2.28; maximum, 2.33; no weights >5 or 10), with an effective sample size of 95.5/110 (48.2 PET, 47.4 angiography) (Table 8). Angiography was not significantly associated with odds of listing (OR, 1.62; 95% CI, 0.54-4.83; p=0.389), time to listing via the landmark approach (HR, 1.18; 95% CI, 0.68-2.06; p=0.549), or one-year MACE (OR, 0.70; 95% CI, 0.16-3.12; p=0.643) (Table 8). Results were essentially unchanged after weight truncation at both percentile levels, and a doubly robust model additionally adjusting for LVEF and dialysis duration yielded a similar estimate (OR, 1.67; 95% CI, 0.48-5.83; p=0.42), concordant with the primary Firth-based result.

**Table 7:** Covariate Balance Before and After Inverse Probability of Treatment Weighting Values are presented as group means for continuous variables or proportions for categorical variables before and after stabilized inverse probability of treatment weighting. Propensity scores were estimated using a reduced model (age and known coronary artery disease (CAD); identified by directed acyclic graph) rather than the full 12-parameter specification, which failed to converge because of limited propensity-score overlap in some covariate strata. Age and CAD, which were included in the propensity model, are well balanced after weighting; residual imbalance remains for dialysis duration, hypertension, and LVEF, which were not included in the propensity model (see Discussion). SMD = standardized mean difference; absolute SMD values <0.1 indicate small standardized differences between groups; PET = positron emission tomography; LVEF = left ventricular ejection fraction; eGFR = estimated glomerular filtration rate.

Covariate	Unweighted, PET	Unweighted, Coronary angiography	Weighted, PET	Weighted, Coronary angiography	SMD Before Weighting	SMD After Weighting
Age	59.80	56.56	58.39	58.15	0.35	0.03
Sex (Female vs. Male)	0.35	0.31	0.32	0.32	0.08	0.00
Body mass index	30.87	30.27	30.39	30.39	0.11	0.00
Dialysis duration	29.58	24.79	31.84	23.28	0.18	0.33
Diabetes mellitus	0.85	0.80	0.81	0.82	0.15	0.01
Hypertension	0.98	0.96	0.99	0.95	0.11	0.22
Coronary artery disease	0.36	0.65	0.50	0.51	0.60	0.01
LVEF	60.31	55.42	59.36	56.52	0.62	0.37
Smoking history	0.29	0.35	0.27	0.29	0.13	0.05
Peripheral artery disease	0.04	0.06	0.05	0.07	0.09	0.08
eGFR	10.36	9.60	10.16	9.69	0.17	0.11

**Figure 4:**
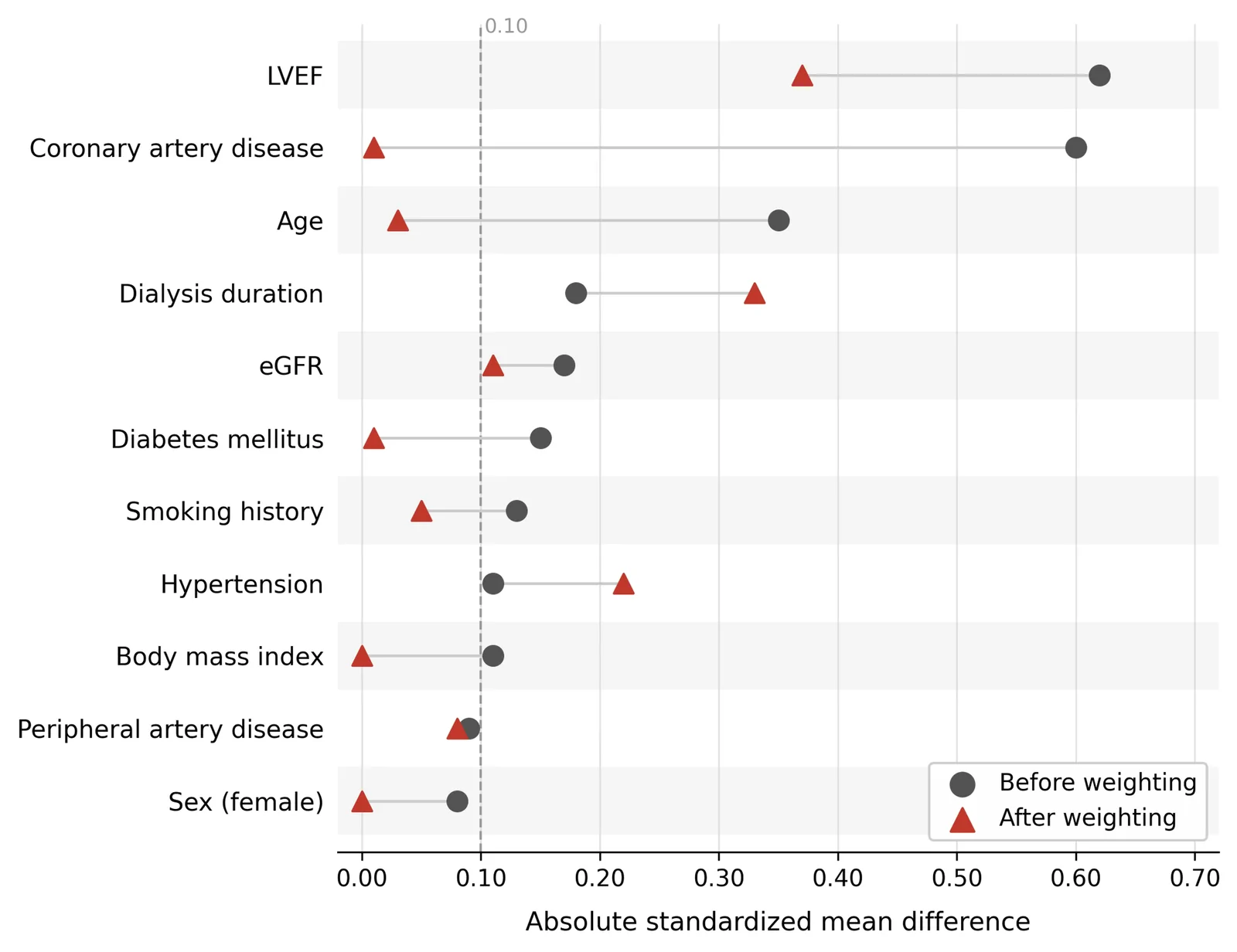
Covariate Balance Before and After Propensity-Score Weighting (Love Plot). Each row represents one covariate included in the propensity model assessment. The x-axis shows the absolute standardized mean difference (SMD). Circles (●) represent the unweighted SMD; triangles (▲) represent the SMD after stabilized inverse probability of treatment weighting (IPTW). The dashed vertical line at SMD = 0.10 indicates the conventional threshold for adequate covariate balance. Age and coronary artery disease (CAD), the two variables included in the propensity model, are balanced essentially exactly after weighting (SMD ≤0.03). Residual imbalance remains for LVEF (SMD = 0.37), dialysis duration (SMD = 0.33), and hypertension (SMD = 0.22); these were not included in the propensity model and were partially addressed in a doubly robust sensitivity analysis (see Results). LVEF = left ventricular ejection fraction; eGFR = estimated glomerular filtration rate; SMD = standardized mean difference.

**Table 8:** Propensity Score-Weighted Associations Between Initial Cardiac Evaluation Modality and Outcomes Propensity score weighting targeted the average treatment effect using stabilized inverse probability-of-treatment weights, estimated from a reduced propensity model (age and known CAD). Estimates compare coronary angiography with PET as the initial cardiac testing modality and use robust (sandwich) standard errors. Propensity scores showed substantial overlap between groups, with observed ranges of 0.215 to 0.785 in the PET group and 0.218 to 0.857 in the angiography group; no weight exceeded 2.33 (no weights >5 or >10), and results were unchanged after truncating weights at the 1st/99th and 5th/95th percentiles. The effective sample size (ESS) was calculated as ESS = (Σw)^2 / Σ(w^2). The time-to-listing estimate uses the landmark analysis restricted to patients not yet listed at their index cardiac test (test date as time origin; see Methods and Results), combined with IPTW, rather than the evaluation-date time origin used elsewhere, because of the temporal-ordering bias described in the Methods and Limitations. A doubly robust model that additionally adjusted for LVEF and dialysis duration yielded a similar waitlisting estimate (OR, 1.67; 95% CI, 0.48-5.83; p=0.42), supporting the robustness of this result to residual imbalance in these two covariates. CI = confidence interval; IPTW = inverse probability of treatment weighting; LVEF = left ventricular ejection fraction; MACE = major adverse cardiovascular events; OR = odds ratio; HR = hazard ratio; PET = positron emission tomography.

Outcome	Unweighted analytic sample, n	Effective sample size, PET	Effective sample size, Angiography	Weighted OR/HR	95% CI	p-value
Listing for transplant (OR)	110	48.2	47.4	1.62	0.54-4.83	0.389
Time to listing, landmark analysis (HR)	55	24.1	23.7	1.18	0.68-2.06	0.549
1-year MACE (OR)	108	47.3	46.5	0.70	0.16-3.12	0.643

Sensitivity analyses, missing data, and cardiac testing pathway

Crossover-exclusion and "ever coronary angiography" analyses remained directionally consistent with the primary results but should be interpreted with caution given the reduced sample sizes. Missingness was <5% for most variables, except for dialysis duration (21/116, 18.1%), and multiple imputation results were consistent with complete-case analyses (Table 9). Among angiography patients, 15/59 (25.4%) had no obstructive disease; 14/59 (23.7%), 10/59 (16.9%), and 12/59 (20.3%) had 1-, 2-, and 3-vessel disease, respectively, and two patients (3.4%) had significant left main disease, as identified by manual chart review (Table 10). Among PET patients, 12/57 (21.1%) were subsequently referred for angiography (median: 65 days; IQR: 35.5-118.0). Crossover frequency was similar (12/57, 21.1% PET; 13/59, 22.0% angiography); patients undergoing additional testing had a numerically longer, nonsignificant time to active listing (511 vs. 272.5 days; p=0.11). Structured data on PET result category, ischemic burden, myocardial blood flow, coronary flow reserve, revascularization timing, procedural complications, and time to cardiology clearance or committee decision were not available in this dataset.

**Table 9:** Missing Data Assessment Complete-case analysis was used for variables with <5% missingness; multiple imputation was used for dialysis duration, which had ≥5% missingness. One additional patient had an indeterminate CAD status and was excluded from the stratified CAD subgroup analysis but is not represented in the CAD missing-data count above. CAD = coronary artery disease; CC = complete-case analysis; MI = multiple imputations by chained equations; N/A = not applicable; LVEF = left ventricular ejection fraction; eGFR = estimated glomerular filtration rate.

Variable	n Missing	% Missing	Handling Method
Age	4	3.4	CC
Sex	2	1.7	CC
Race	2	1.7	CC
Body mass index	3	2.6	CC
Dialysis duration	21	18.1	MI
Diabetes mellitus	3	2.6	CC
Hypertension	3	2.6	CC
Smoking history	4	3.4	CC
Pack-years	0	N/A	N/A
Coronary artery disease	2	1.7	CC
LVEF	3	2.6	CC
eGFR	3	2.6	CC
Peripheral artery disease	3	2.6	CC
Cerebrovascular disease	0	N/A	N/A

**Table 10:** Cardiac Testing Findings, Revascularization, and Cross-Modality Referral by Initial Cardiac Evaluation Modality Vessel-disease categories and the significant left main disease count are derived from the "number of affected vessels" field and, for left main disease, from manual review of free-text angiography reports; these were not structured, prespecified fields and should be considered approximate. Six of 59 angiography patients (10.2%) lacked sufficient structured data to assign a vessel-disease category and are not represented in those rows; percentages for vessel-disease categories therefore do not sum to 100%. Structured data were not available for the PET result category (normal, abnormal, or high-risk), ischemic burden, myocardial blood flow, coronary flow reserve, immediate versus later timing of revascularization, or procedural complications (e.g., contrast-associated kidney injury, bleeding, or hospitalization); these are discussed in the Limitations. ᵃAmong the 12 PET patients who crossed over to angiography, one patient was excluded due to an implausible negative interval, likely a data-entry error. ᵇIdentified by manual review of free-text angiography reports, not a structured field; may undercount or overcount true left main disease. ᶜAs reported in Table 5. CAD = coronary artery disease; IQR = interquartile range; N/A = not applicable; PET = positron emission tomography.

Variable	PET (n = 57)	Coronary angiography (n = 59)
Referred for angiography after PET, n/N (%)	12/57 (21.1)	N/A
Time from PET to subsequent angiography, days, median (IQR)ᵃ	65.0 (35.5-118.0)	N/A
No obstructive CAD (0 vessels), n/N (%)	N/A	15/59 (25.4)
1-vessel disease, n/N (%)	N/A	14/59 (23.7)
2-vessel disease, n/N (%)	N/A	10/59 (16.9)
3-vessel disease, n/N (%)	N/A	12/59 (20.3)
Diffuse/≥4-territory disease, n/N (%)	N/A	2/59 (3.4)
Significant left main disease (≥50% stenosis), n/N (%)ᵇ	N/A	2/59 (3.4)
Underwent coronary revascularization, n/N (%)ᶜ	9/57 (15.8)	13/59 (22.0)
Crossed over to the other modality, n/N (%)	12/57 (21.1)	13/59 (22.0)

## Discussion

Summary of principal findings

In this cohort of 116 kidney transplant candidates, initial coronary angiography was associated with higher odds of listing in the primary model, but this did not reach significance. The reduced propensity-weighted model found no significant association between testing modality and waitlisting, consistent with the Firth-based analysis. The apparent unadjusted PET advantage in time to listing was substantially affected by reverse temporal ordering: over half of the PET and two-fifths of the angiography patients were already listed before their index test. In a landmark analysis that corrected for this, time to listing did not differ overall, though angiography was associated with faster listing among patients with no known CAD specifically (HR, 2.58; 95% CI, 1.08-6.16), an exploratory finding given the small subgroup. Cardiovascular outcomes showed no statistically significant difference, but with only eight MACE events, the study was underpowered and could not establish comparable safety.

Comparison with literature and clinical implications

Current guidance supports a risk-stratified approach to cardiac evaluation rather than a uniform strategy [3,4]. Known CAD status differed markedly by modality in our cohort (35/56, 62.5% of no-known-CAD patients received PET; 37/57, 64.9% of known-CAD patients received angiography), indicating the initial test functioned as a marker of clinical indication rather than an interchangeable, randomly assigned choice. This reframes the central comparison as one between clinician-selected pathways for different indications, rather than as an equivalence between testing strategies.

Positron emission tomography may aid in detecting both focal and diffuse coronary disease through noninvasive functional assessment and quantification of myocardial blood flow and coronary flow reserve. However, its availability varies, and its first-line role remains incompletely defined [2-6]. Angiography allows direct anatomic assessment and, when indicated, revascularization [3,7,8], but the routine use of invasive testing in asymptomatic advanced kidney disease remains controversial. In the ISCHEMIA-CKD trial, an initial invasive strategy did not reduce death or myocardial infarction versus a conservative strategy [9], and subsequent analyses showed no clear mortality advantage with an invasive approach [11]. Recent perioperative and multimodality imaging guidance similarly favors selective, risk-based testing over routine invasive evaluation [12,13].

The no-known-CAD subgroup most closely resembles the American Heart Association (AHA) statement's description of screening for previously unknown disease.

Consistent with the AHA statement's observation that screening asymptomatic candidates has not been shown to improve clinical outcomes, this exploratory subgroup finding requires independent confirmation before it can inform practice and should not be interpreted as evidence that either modality is preferable in this population.

Strengths and limitations

Strengths include a clinically important, real-world question; a clinically meaningful primary outcome, given the established survival benefit of transplantation over dialysis [14,15]; classification by the initial cardiac test performed; and multiple complementary analytic approaches (multivariable regression, Cox modeling, IPTW, crossover exclusion, and doubly robust modeling).

Several limitations warrant emphasis. First, the primary time-to-event analyses originally used the evaluation date as the time origin despite the exposure being defined by a later test; this was corrected via the landmark analysis. Second, the initial test was clinician-selected based on known CAD status, meaning the overall comparison substantially reflects confounding by indication; symptom status and prior revascularization details were unavailable, and the known-CAD subgroup comprised only six PET patients. Third, the retrospective, single-center design and clinician-driven test selection introduce further potential selection and information bias. Fourth, unmeasured confounding (e.g., frailty, symptom burden, socioeconomic status, provider preference) cannot be excluded; some covariates were unstable in the full model, consistent with quasi-complete separation, and were therefore excluded from the primary specification. Fifth, the original 12-covariate propensity model did not converge; the corrected reduced model achieved good overlap and an effective sample size but left residual imbalance in LVEF and dialysis duration, which a doubly robust model partially addressed. Sixth, crossover between modalities, modest sample size, wide confidence intervals, and low event rates limited power, particularly for cardiovascular outcomes. Seventh, a structured reason for nonlisting was not captured, so the extent to which cardiac findings versus other factors (frailty, malignancy, infection, obesity, adherence, psychosocial factors, or insurance and administrative barriers) drove nonlisting cannot be determined; one nonlisted patient was later found listed and transplanted at another center, indicating outside-center outcomes were incompletely captured. Eighth, detailed cardiac test results and downstream pathway data (ischemic burden, flow reserve, revascularization timing, procedural complications) were not systematically available and should be prioritized in future data collection. Ninth, the CAD-stratified analyses were exploratory and underpowered. A formal modality-by-CAD interaction test could not be reliably reconstructed from the archived analytic dataset; therefore, the subgroup findings should not be interpreted as evidence of effect modification. Minor discrepancies between baseline descriptive CAD coding and the CAD-stratified analytic classification could not be fully reconciled from the archived dataset. Tenth, the specific parameters of the multiple imputation procedure used for dialysis duration (number of imputed datasets, imputation model variables, iteration count, and pooling method) were not recorded in the retained analysis materials and could not be verified; the imputed and complete-case analyses were nonetheless concordant. In addition, uncertainty from the original abstraction rule for waitlisting dates, differences in analytic denominators due to missing dates and covariate data, and the inability to reproduce selected historical inferential statistics from the archived analytic dataset limit reproducibility and reduce confidence in the precision of individual estimates. Accordingly, these findings should be interpreted as hypothesis-generating rather than definitive. Finally, the single-center design limits generalizability, as test availability and workflows may differ elsewhere.

Future directions

Prospective multicenter studies using time-appropriate analytic methods (landmark or time-dependent exposure designs) are needed to compare cardiac evaluation pathways, incorporating symptom status, specific testing indications, frailty, and socioeconomic factors. The present findings may help generate hypotheses for such studies, but do not establish which initial cardiac testing strategy should be preferred.

## Conclusions

In this retrospective cohort, initial cardiac testing was clinician-selected and strongly associated with known CAD status, resulting in substantial potential for confounding by indication; therefore, the findings are best interpreted as associations between clinician-selected testing pathways rather than comparative-effectiveness estimates for interchangeable strategies. Across the primary landmark analysis and complementary Firth, IPTW, and doubly robust analyses, no statistically significant overall association between testing modality and subsequent waitlisting was demonstrated. The CAD-stratified finding among candidates without known CAD was exploratory and, in the absence of a reproducible formal interaction test, should not be interpreted as evidence of effect modification. The limited number of cardiovascular events also precludes conclusions regarding comparative safety. Given the observational design, residual confounding, and modest sample size, these findings are hypothesis-generating and do not establish the superiority, equivalent safety, or preferred clinical use of either PET or coronary angiography. Adequately powered, prospective multicenter studies using time-appropriate analytic methods and standardized testing indications are needed to compare initial cardiac evaluation strategies in kidney transplant candidates.
